# Mucus-penetrating microbiota drive chronic low-grade intestinal inflammation and metabolic dysregulation

**DOI:** 10.1080/19490976.2025.2455790

**Published:** 2025-01-26

**Authors:** Melissa C. Kordahi, Noëmie Daniel, Andrew T. Gewirtz, Benoit Chassaing

**Affiliations:** a Microbiome-Host Interactions, Institut Pasteur, Université Paris Cité, INSERM U1306, CNRS UMR6047, Paris, France; b Mucosal microbiota in chronic inflammatory diseases, INSERM U1016, CNRS UMR8104, Université Paris Cité, Paris, France; c Institute for Biomedical Sciences, Centre for Inflammation, Immunity and Infection, Digestive Disease Research Group, Georgia State University, Atlanta, GA, USA; d CHRU Nancy, IHU Infiny, Nancy, France

**Keywords:** Microbiota, mucus, encroachment, inflammation, metabolic deregulations

## Abstract

Metabolic syndrome is, in humans, associated with alterations in the composition and localization of the intestinal microbiota, including encroachment of bacteria within the colon’s inner mucus layer. Possible promoters of these events include dietary emulsifiers, such as carboxymethylcellulose (CMC) and polysorbate-80 (P80), which, in mice, result in altered microbiota composition, encroachment, low-grade inflammation and metabolic syndrome. While assessments of gut microbiota composition have largely focused on fecal/luminal samples, we hypothesize an outsized role for changes in mucus microbiota in driving low-grade inflammation and its consequences. In support of this notion, we herein report that both CMC and P80 led to stark changes in the mucus microbiome, markedly distinct from those observed in feces. Moreover, transfer of mucus microbiota from CMC- and P80-fed mice to germfree mice resulted in microbiota encroachment, low-grade inflammation, and various features of metabolic syndrome. Thus, we conclude that mucus-associated bacteria are pivotal determinants of intestinal inflammatory tone and host metabolism.

## Introduction

Mammalian intestinal mucosal surfaces harbor a dense and diverse community of microorganisms, referred to as microbiota.^1^ Most of these microbes are anaerobic bacteria that benefit the host. Such benefits include mediating digestive processes, limiting pathogen colonization, and promoting immune system development.^1^ On the other hand, the microbiota produces a myriad of Microbe-Associated Molecular Patterns (MAMPs) that hold the potential to activate various receptors of the immune system in a way that can promote chronic intestinal inflammation.^2^ In order to maintain intestinal homeostasis, various mechanisms are in place in order to protect the host against over-activation by its endogenous microbiota. These include intestinal goblet cells which secrete large quantities of mucin that form a protective layer of gel-like mucus over the epithelial lining.^3^ This mucus layer is composed of two distinct strata: the outer layer, which is colonized with bacteria, and the inner layer which is mostly resistant to bacterial colonization, forming a protected nearly sterile zone adjacent to the epithelial surface. Accumulating data suggest that the integrity of this inner mucus barrier is one of the first lines of protection of the gastrointestinal tract against its intestinal microbiota. For example, mice lacking Muc2, which is the predominant mucin in the colon, are unable to maintain the microbiota at a proper distance from the epithelial lining and, consequently, develop severe chronic intestinal inflammation.^4,5^

Diet strongly influences how the intestinal microbiota interacts with the gastrointestinal tract.^4^ For example, consumption of diets low in fiber and high in ultra-processed foods is associated with increased risk of developing chronic inflammatory diseases such as Inflammatory Bowel Disease (IBD) and metabolic syndrome.^6–10^ We have shown that some dietary emulsifiers, which are frequently added to many ultra-processed foods to improve organoleptic properties and extend shelf-life, alter the microbiota in a way that promotes chronic intestinal inflammation and metabolic dysregulations.^11,12^ Emulsifier-induced alterations in microbiota include changes in its species composition and its localization, with emulsifier consumption driving microbiota to enter into the normally near-sterile inner mucus layer.^11,13^ Such microbiota encroachment is a key feature observed in an array of chronic inflammatory disorders including Crohn’s disease and type 2 diabetes,^14,15^ suggesting an important role played by the mucus-associated microbiota in driving intestinal inflammation.

Here, we investigated to which extent mucus-associated bacteria that encroach within the inner intestinal mucus layer are playing a direct role in driving chronic intestinal inflammation and associated metabolic dysregulations in their host. We report that emulsifier consumption not only changes microbiota localization, but also strongly alters the composition of mucus-associated microbiota. Furthermore, transplantation of mucus-associated microbiota into germ-free mice was sufficient to transfer microbiota encroachment as well as subacute intestinal inflammation and metabolic dysregulations. Thus, alteration in mucus microbiota composition is a primary event in driving microbiota encroachment, chronic low-grade intestinal inflammation and its downstream detrimental consequences on host metabolism.

## Results

### Dietary emulsifiers consumption reproducibly drives chronic low-grade intestinal inflammation and metabolic dysregulations

**Figure 1.**
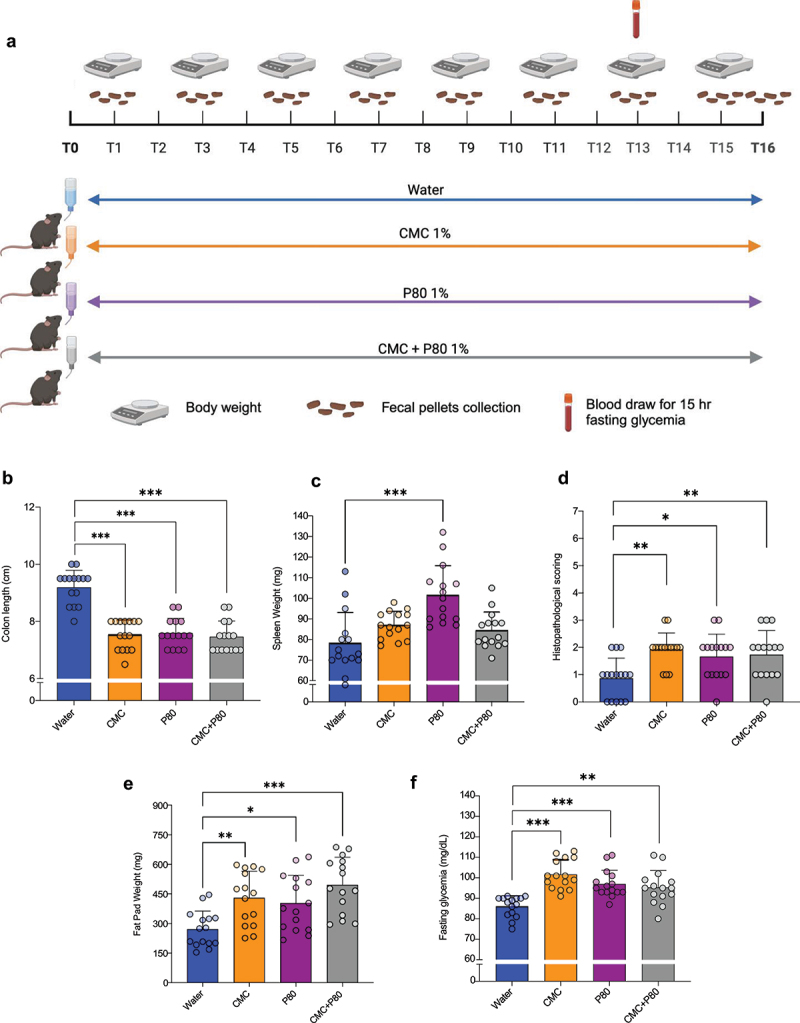
Dietary emulsifiers consumption reproducibly drives chronic low-grade intestinal inflammation and metabolic dysregulations. (a) Schematic representation of the experimental design used. Mice were exposed to drinking water (blue) containing 1.0% of CMC (orange), P80 (purple), or CMC + P80 (gray) for 16 weeks. (b) Colon length, (c) spleen weight (d) histopathological scoring of intestinal inflammation on H&E-stained colonic sections, (e) epididymal fat pad weight and (f) 15-hours fasting blood glucose level. Data are represented as means ± SD. *N* = 15. For bar graphs, statistical analyses were performed using a one-way ANOVA followed by a Bonferroni post hoc test and significant differences were recorded as follows: *p < 0.05, **p < 0.01, ***p < 0.001. ANOVA, analysis of variance; CMC, carboxymethylcellulose; P80, polysorbate 80.

The central goal of this study was to define the impacts of emulsifier consumption on mucus microbiota and examine the role played by this specific community in driving intestinal inflammation and its consequences. This necessitated that we first reproduce previously reported impacts of such compounds on the microbiota and host.^11^ For this purpose, WT C57Bl/6 mice were administered carboxymethylcellulose (CMC) or polysorbate-80 (P80) emulsifiers, or a combination of both emulsifiers (CMC + P80 group), *via* drinking water (Figure 1a). As expected, emulsifier consumption drove chronic low-grade intestinal inflammation, as evidenced by colon shortening, a macroscopical marker of intestinal inflammation in mice, spleen enlargement, and histopathological analysis of colon specimens (Figure 1b-d).

Metabolic dysregulations are associated with and promoted by chronic low-grade inflammation. Thus, we next examined whether emulsifiers-induced low-grade inflammation was associated with metabolic dysregulation. In further accord with previous observations, both CMC and P80 emulsifiers consumption resulted in marked increase in fat deposition, as measured by peri-epididymal fat pad weight (Figure 1e), without an associated increase in the overall body weight (Figure S1A). Moreover, CMC or P80 consumption significantly impaired glycemic control as assessed by measuring fasting blood glucose concentration, revealing a significant increase compared to water-treated mice (Figure 1f). Interestingly, we did not observe an aggravated phenotype when treating mice with a combination of CMC and P80, suggesting that CMC and P80 act through similar mechanisms to induce low-grade intestinal inflammation and metabolic dysregulations.

### Dietary emulsifiers consumption reproducibly drives intestinal microbiota alterations, increasing microbiota pro-inflammatory potential and promoting microbiota encroachment

**Figure 2.**
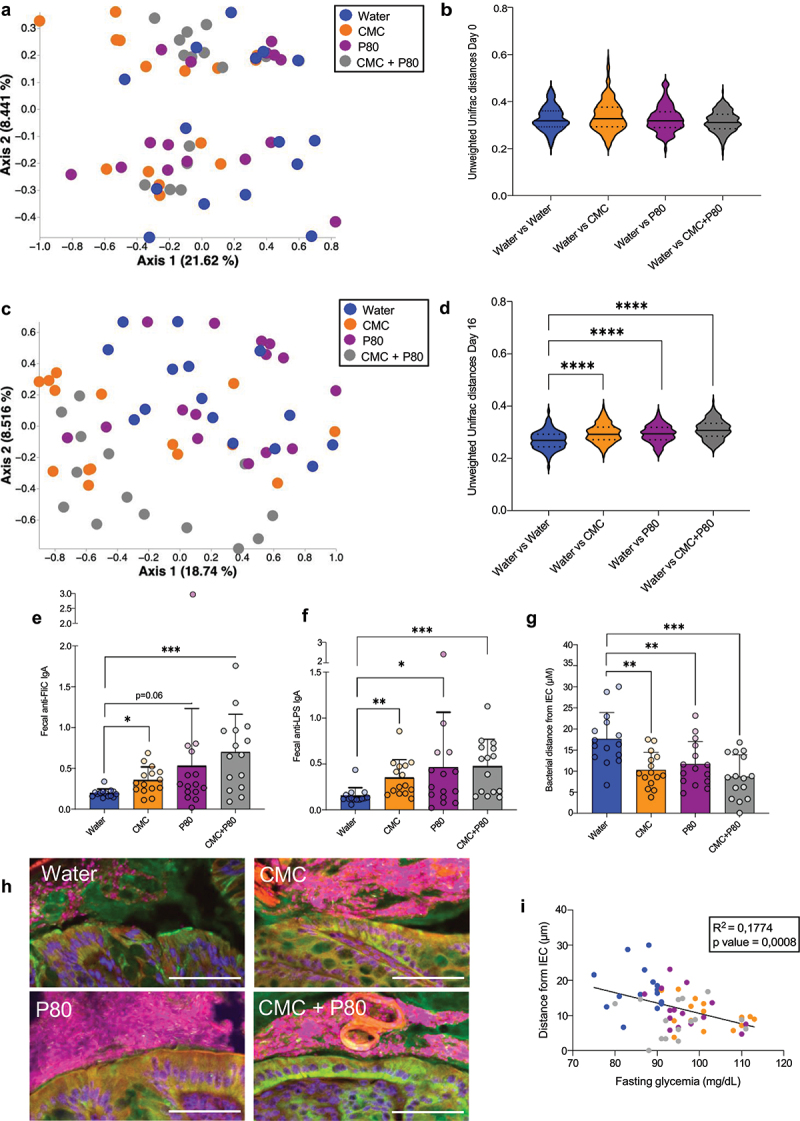
Dietary emulsifiers consumption reproducibly drives intestinal microbiota alterations, increasing microbiota pro-inflammatory potential and promoting microbiota encroachment. Bacterial DNA was extracted from feces samples and subjected to 16S rRNA gene sequencing on week 0 and week 16 of the protocol. (a, c) Principal coordinates analysis (PcoA) of the unweighted Unifrac distance matrices of microbiota composition at week 0 (a) and week 16 (c) and assessed by 16S rRNA gene sequencing. Each dot represents an individual animal and are colored by experimental group (blue, Water; orange, CMC; purple, P80; grey, CMC + P80). (b, d) unweighted Unifrac distances at week 0 (b) and week 16 (d) of the protocol separating water-treated mice and water- (blue), CMC- (orange), P80- (purple) or CMC + P80- (gray) treated mice. (e-f) fecal levels of anti-flagellin (e) and anti-lps (f) IgA. (g) Colonic biopsies were subjected to immunostaining paired with fluorescent *in situ* hybridization (FISH) followed by confocal microscopy analysis of microbiota localization. (h) Representative pictures obtained from 5 biological replicates. MUC2, green; actin, purple; bacteria, red; and DNA, blue. Scale bar, 50 µm (i) distances of the closest bacteria to intestinal epithelial cells (IEC), measured using 5 high-powered fields per mouse and plotted versus fasting blood glucose concentration. Data are represented as means ± SD. *N* = 15. For bar graphs, statistical analyses were performed using a one-way ANOVA followed by a Bonferroni post hoc test and significant differences were recorded as follows: *p < 0.05, **p < 0.01, ***p < 0.001. ANOVA, analysis of variance; CMC, carboxymethylcellulose; P80, polysorbate 80.

The intestinal microbiota has been previously observed to be a central actor in mediating emulsifiers-induced chronic intestinal inflammation and metabolic dysregulations.^11,16^ Use of 16S rRNA sequencing followed by principal coordinate analysis (PCoA) of the unweighted Unifrac distances revealed that, while mice harbored homogeneous baseline microbiotas (Week 0, Figure 2a-b), 16 weeks of exposure to CMC and/or P80 resulted in treatment-based microbiota clustering (Week 16, Figure 2c-d), especially in mice subjected to CMC or CMC+P80 regimen. These data confirm previous observations that both CMC and P80 hold the potential to markedly shift the intestinal microbiota composition. Of note, we observed different clustering between CMC-treated and P80-treated mice, suggesting compound-specific effects on microbiota composition. Moreover, mice treated with the combination of both CMC and P80 emulsifiers harbored compositionally similar microbiota compared to mice exposed to CMC only, suggesting a dominant effect of CMC on altering the intestinal microbiota compared to P80 (Figure 2c-d).

Analysis of the alpha diversity through calculation of the Simpson index revealed no significant differences between the various groups (Figure S1B). A taxonomical bar plot representation of the relative abundances of the various taxa present, together with a MaAsLin2 (Microbiome Multivariable Associations with Linear Models) analysis were next applied to visualize and identify features significantly impacted by emulsifiers exposure compared to water-treated animals. Features belonging to the *Enterousia* genus were increased in both groups consuming dietary emulsifier. Features belonging to the *Bacteroides_H, CAG.95, Lawsonibacter* and *Faecimonas* genera were found to be significantly increased by P80 consumption, while features belonging to the *Acetitomaculum, Lachnospira*, and *Faecousia* genera as well as features belonging to the Gastranaerophilaceae, Ruminococcaceae, and UBA660 families were found increased in mice consuming CMC, further suggesting compound-dependent effects of emulsifiers on the intestinal microbiota. Features belonging to the *Clostridum_AP*, *Massilioclostridium, Phocea*, CAG.314, *Eubacterium_S, Sporofaciens, Ventrimonas*, Erysipelatoclostridium, Ligilactobacillus, and CAG.605 genera were significantly increased in mice consuming CMC and/or the combination of CMC and P80. Moreover, compared to the water-treated group, mice consuming any type of emulsifiers had a decrease in features belonging to the UBA3263, *Robinsoniella*, Eubacterium_R and UBA_644 genera as well as to features belonging to the Muribaculaceae family. Mice consuming P80 alone or in combination with CMC had a depletion in a feature belonging to the *Avispirillum* genus and in a feature belonging to the CAG.74 family, whereas mice consuming CMC and/or the combination of CMC and P80 had a depletion in features belonging to the *Alloprevotella, Prevotella, Paramuribaculum*, UBA7173, *Ruminococcus_C_58660*, UBA946, *Dubosiella, Turicimonas*, and *Akkermansia* genera (Figure S1C-D). Such alterations at the compositional level were previously reported to be associated with an increase in the microbiota pro-inflammatory potential, characterized by an elevation in bioactive levels of fecal flagellin (FliC) and lipopolysaccharide (LPS) and an associated increased levels of fecal anti-flagellin and anti-LPS antibodies.^11,12,16^ Concomitantly, we observed here a significant elevation in fecal anti-flagellin as well as fecal anti-LPS immunoglobulin A (IgA) in mice consuming emulsifiers CMC and/or P80 compared to water-treated mice (Figure 2e-f).

Another consequence of dietary emulsifier consumption is the promotion of microbiota encroachment within the normally near-sterile inner mucus layer. Hence, we next used immunostaining of mucins and bacteria localization by FISH, followed by confocal-based determination of epithelium/microbiota distance,^11,12,16^ revealing that consumption of CMC and/or P80 emulsifiers is sufficient to induce severe microbiota encroachment, with the average microbiota/epithelium distance being reduced from 17,67 μm ±6,24 μm in water-treated mice to 10,29 μm ±4,16 μm in CMC-treated mice, 11,67 μm ±5,38 μm in P80-treated mice, and 8,71 μm ±5,18 μm in CMC+P80-treated mice (Figure 2g-h). We also observed a negative correlation between the epithelium/microbiota distance and the fasting glycemia levels measured (Figure 2i), reinforcing a potential causal link between microbiota encroachment and metabolic dysregulations promoted by chronic low-grade intestinal inflammation, as previously reported in both mice and humans.^11,14^ Altogether, these data highlight that the consumption of dietary emulsifiers induces stark and reproducible alterations in microbiota composition, localization, and interaction with the intestinal epithelial layer in a way that is associated with the promotion of chronic low-grade intestinal inflammation and metabolic dysregulations.

### Dietary emulsifiers consumption alters microbiota composition at the mucosal level

**Figure 3.**
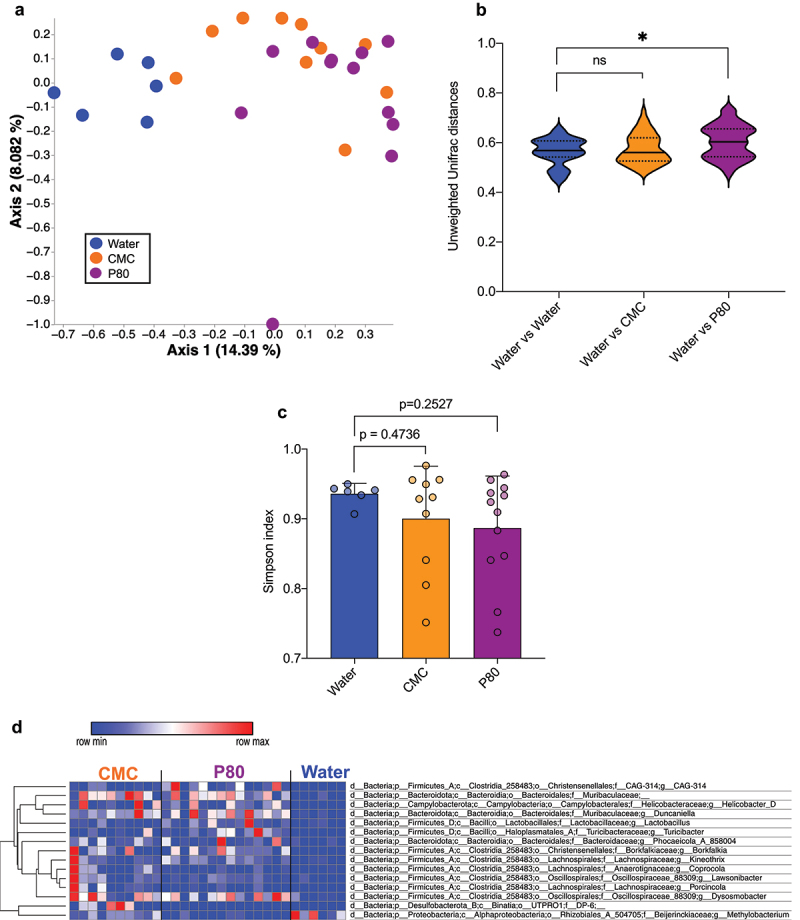
Dietary emulsifiers consumption alters microbiota composition at the mucosal level. Mucus-associated DNA was extracted from laser capture micro-dissected inner mucus layer and subjected to 16S rRNA gene amplification and sequencing. (a) Principal coordinates analysis (PCoA) of the unweighted Unifrac distance matrix of microbiota composition assessed by 16S rRNA gene sequencing. Each dot represents an individual animal and are colored by experimental group (blue, Water; orange, CMC; purple, P80). (b) Unweighted Unifrac distances at week 16. (c) Simpson diversity index at week 16. (d) Significantly differentially abundant features were identified using microbiome multivariable associations with linear models (MaAslin 2) approach, presented as heatmaps. In bar graphs, data are represented as means ± SD. *N* = 5–15. Statistical analyses were performed using a one-way ANOVA followed by a Bonferroni post-hoc test and significant differences were recorded as follows: *p < 0.05, **p < 0.01, ***p < 0.001. ANOVA, analysis of variance; CMC, carboxymethylcellulose; P80, polysorbate 80.

We hypothesized that emulsifiers might not only change the localization of the microbiota within the mucus layer, but also its composition. Hence, we analyzed microbiota composition within the inner mucus layer *via* our previously described laser-capture microdissection method.^17^ Such an approach allows to interrogate microbiota composition at the mucosal side *via* laser capture microdissection of the inner mucus layer followed by DNA extraction and 16S rRNA sequencing.^17^ Application of this technique revealed significant alterations of this mucus-associated ecosystem following dietary emulsifiers consumption, with the observation of clearly distinct clustering induced by CMC or P80 consumption compared to the water-treated control group (Figure 3a). This was further highlighted by significantly increased unweighted Unifrac distances between the water-treated group and the P80-treated group, as reported Figure 3b. Alpha diversity (Simpson index) analysis revealed similar microbiota richness between all groups (Figure 3c). MaAsLin2 (Microbiome Multivariable Associations with Linear Models) analysis was next applied in order to identify mucus-associated microbiota members significantly impacted in their relative abundance by emulsifier consumption. In both CMC- and P80-treated mice, features belonging to the *Kineothrix* genus and the Muribaculaceae family were significantly increased compared to water-treated control mice. In P80-treated mice, we observed a depletion in a feature belonging to the *Methylobacterium* genus and a significant increase in features belonging to the *Helicobacter_D, Borkfalia*, CAG-314, *Lactobacillus*, and *Turicibacter* genera, whereas in CMC-treated mice, we observed an increase in features belonging to the *Phocaeicola_A_858004*, *Coprocola, Duncaniella, Dysosmobacter, Lawsonibacter, Porcincola*, and *DP-6* genera (Figure 3d).

### Transplantation of mucus-associated microbiota is sufficient to transfer microbiota encroachment and associated chronic intestinal inflammation and metabolic dysregulations in germ-free recipient naive mice

**Figure 4.**
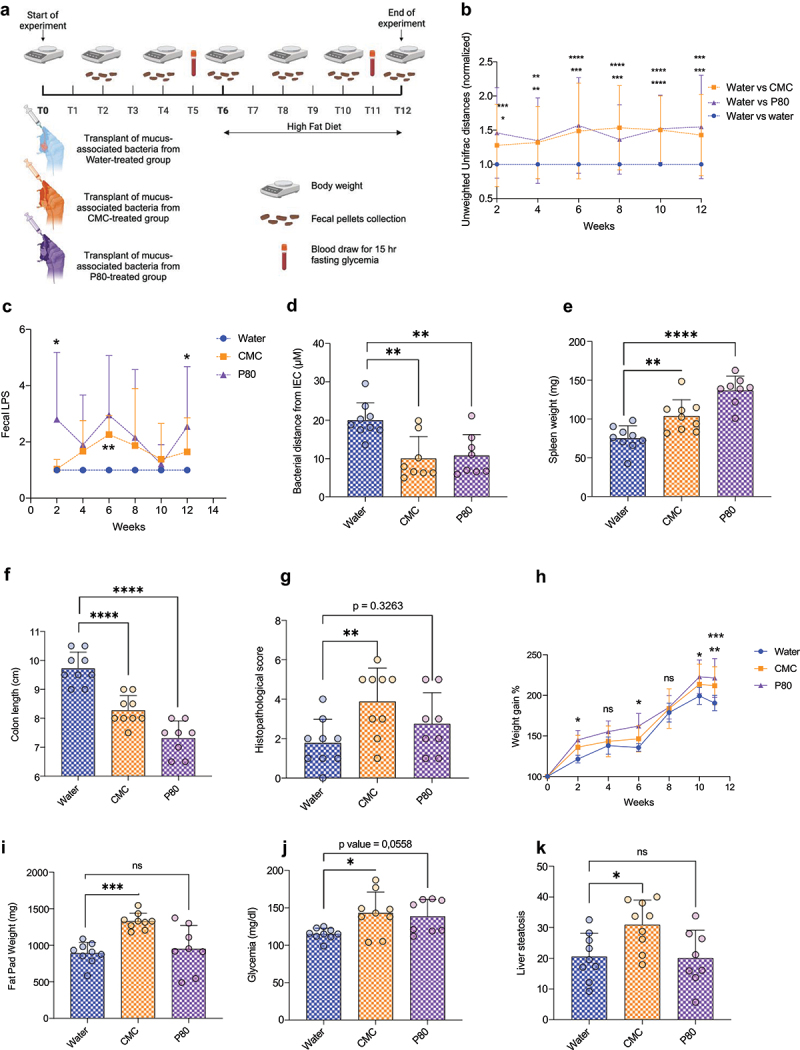
Transplantation of mucus-associated microbiota is sufficient to transfer microbiota encroachment and associated chronic intestinal inflammation and metabolic dysregulations in germ-free recipient naive mice. (a) Schematic representation of the experimental design used. Germ-free mice were inoculated with mucosal biopsy homogenates from mice having consumed water (blue), CMC (orange) or P80 (purple). Body weight was measured over time and feces were collected every other week. (b) Longitudinal analysis of the unweighted unifrac distance matrix of the intestinal microbiota assessed by 16S rRNA gene sequencing. Unweighted unifrac distances were normalized to water-water distances, defined as 1. (c) Longitudinal analysis of bioactive fecal LPS levels. Fecal LPS levels were normalized to levels in the control group, defined as 1. (d) Colon was subjected to immunostaining paired with fluorescent *in situ* hybridization (FISH) followed by confocal microscopy analysis of microbiota localization. Distances of closest bacteria to intestinal epithelial cells (IEC) per condition over five high-powered fields per mouse. (e) Spleen weight, (f) colon length, and (g) histopathological scoring of intestinal inflammation on H&E-stained colonic sections. (h) Body weight gain over time, (i) epididymal fat pad weight, (j) 15-hours fasting blood glucose levels, and (k) liver steatosis scoring. Data are represented as means ± SD. *N* = 8–9. For bar graphs, statistical analyses were performed using a one-way ANOVA followed by a Bonferroni post hoc test and significant differences were recorded as follows: *p < 0.05, **p < 0.01, ***p < 0.001, ****p < 0.0001. ANOVA, analysis of variance; CMC, carboxymethylcellulose; P80, polysorbate 80.

We next performed microbiota transplants to determine whether mucus-associated microbiota observed in emulsifiers-treated mice played a role in driving low-grade chronic intestinal inflammation and associated metabolic dysregulations. Colonic biopsies were collected from water-, CMC- or P80-treated mice, as presented Figure 1a, thoroughly washed in PBS and homogenized in PBS containing 30% glycerol before being stored at − 80°C. These homogenates were then administered to germ-free WT mice (Figure 4a). After a microbiota stabilization period of 6 weeks, reported to be sufficient to drive mucus layer homeostasis post-colonization,^18^ mice were fed a High-Fat Diet (HFD) for an additional 6 weeks in order to investigate susceptibility to diet-induced obesity and metabolic dysregulations (Figure 4a). Use of 16S rRNA sequencing followed by principal coordinate analysis (PCoA) of the unweighted Unifrac distances revealed that transplanted mice exhibited clear group-based microbiota clustering throughout the experiment, despite not being directly exposed to CMC nor P80 dietary emulsifiers (Figure S2A-F). Longitudinal quantification of the unweighted Unifrac distances between groups indeed reported stark and long-lasting compositional alterations between mice having received mucus-associated microbiota from CMC- or P80-treated mice compared to mice having received mucus-associated microbiota from water -treated mice (Figure 4b). This observation suggests that both CMC and P80 consumption drives a marked shift in the mucus-associated microbiota composition, which can be stably transferred into germ-free recipient mice not directly consuming such additives (Figure 4b and S2 A-F). A taxonomical bar plot representation of the relative abundances of the various taxa present, together with a MaAsLin2 analysis revealed that mice transplanted with mucus-associated microbiota from CMC- or P80-treated mice displayed significant alterations in various features compared to mice transplanted with mucus-associated microbiota from water-treated mice. In mice transplanted with mucus-associated microbiota from P80-consuming mice, we observed significant increases in features from the *Clostridium_T, Lawsonibacter*, and *Clostridium_AQ* genera, alongside a notable depletion in features belonging to the Oscillospiraceae_88309 family and the *Anaerotruncus, Lactobacillus*, and *Limosilactobacillus* genera. Similarly, there was a significant reduction in features associated with the *Alloprevotella*, UBA7173, *Acetatifactor, Blautia_A_141780*, and *Copromonas* genera in mice transplanted with mucus-associated microbiota from either CMC or P80-consuming mice compared to those transplanted with microbiota from the water control group. Additionally, an increase in features related to the Lachnospiraceae and Coprobacillaceae families, as well as the *Bacteroides_H, CAG.85, Anaerostipes, Robinsoniella, Acutalibacter, Pseudobutyricicoccus, Anaerotruncus, Angelakisella, Massilioclotridium*, and *Clostridioides_A* genera, were observed in mice transplanted with mucus-associated microbiota from CMC- and/or P80-consuming mice. Concurrently, there was a significant decrease in features related to the *Enterocloster, Parabacteroides_B_862066, Dysosmobacter*, and *Dubosiella* genera (Figure S3A-B). Assessment of alpha diversity using the Simpson index revealed a decreased richness in the group transplanted with mucus-associated microbiota from CMC and P80-consuming mice at 6 weeks post-transplantation, a difference which vanished when mice were switched to a HFD regimen (Figure S3C).

We next investigated functional parameters of the intestinal microbiota in these recipient mice, including bioactive levels of fecal flagellin and LPS as well as microbiota encroachment. While we did not observe significant alterations in the fecal levels of bioactive flagellin (data not shown), fecal levels of LPS were significantly elevated in mice colonized with mucus-associated microbiota collected from emulsifiers-treated mice compared to the control group (Figure 4c). These results suggest that both compositional and functional aspects of emulsifiers-induced dysbiosis were transplanted into recipient mice following mucus-associated microbiota gavage. Further supporting this notion, we next observed that mice transplanted with mucus-associated microbiota from emulsifiers-treated mice harbored microbiota encroachment compared to the control group (Figure 4d and Figure S3D). More specifically, the average bacteria/epithelium distance was reduced from 20,01 μm ±4,53 μm in mice transplanted with mucus-associated microbiota collected from water-treated mice to 10,02 μm ±5,74 μm and 10,84 μm ±5,42 μm in mice transplanted with mucus-associated microbiota collected from CMC- or P80-treated mice, respectively (Figures 4d and S3D).

Microbiota encroachment is associated with low-grade chronic intestinal inflammation as well as metabolic dysregulations, in both mice models and humans,^11,14,19,20^ although whether the encroaching microbiota is a cause and/or consequence of these events has yet to be elucidated. We observed that mice transplanted with mucus-associated microbiota from emulsifiers-treated mice displayed both low-grade intestinal inflammation and, furthermore, exhibited increased susceptibility to diet-induced metabolic dysregulations. More specifically, a significant spleen enlargement and colon shortening were observed in mice transplanted with mucus-associated microbiota collected from emulsifiers-treated mice compared to mice transplanted with mucus-associated microbiota collected from water-treated mice (Figure 4E-F). A significant increase in histopathological scoring performed on H&E-stained colonic sections was observed in mice transplanted with mucus-associated microbiota collected from CMC-consuming mice. In addition, we observed that mice transplanted with mucus-associated microbiota collected from emulsifiers-treated mice harbored a significantly increased susceptibility to metabolic dysregulations induced by HFD consumption, as highlighted by significantly increased body weight gain over the course of the experiment (Figure 4H). Moreover, mice transplanted with mucus-associated microbiota collected from CMC-consuming mice exhibited significantly increased fat deposition (Figure 4I), impaired glycemic control (Figure 4J), as well as increased liver steatosis (Figure 4K). Collectively, these data argue that mucus-invading bacteria hold the potential to initiate low-grade intestinal inflammation and associated metabolic dysregulations.

## Discussion

Intestinal microbiota alterations are associated with various chronic diseases, including IBD, colorectal cancer, and metabolic dysregulations. While much of the focus has been on the fecal microbiome, recent studies suggest that mucus-associated bacteria might play a pivotal role in driving such chronic conditions.^21–24^ Using a well-characterized model of emulsifier-induced microbiota encroachment,^11^ we report here that mucus-associated microbiota subpopulations can directly induce chronic low-grade intestinal inflammation and downstream metabolic dysfunctions when transplanted into germ-free recipient naïve mice. These findings imply that the mucus-associated ecosystem is sufficient to drive phenotypic expression of microbiota encroachment.^11^

In recent decades, consumption of food additives, such as dietary emulsifiers, has increased steadily due to their widespread use in industrialized food production.^10^ Our data, along with findings from other groups, indicate that emulsifiers disturb the host–microbiota relationship, resulting in a microbiota with enhanced mucinophylic and pro-inflammatory activity that promotes intestinal inflammation and subsequent metabolic dysregulation.^11,16^ Here, we observed that the combined use of carboxymethylcellulose (CMC) and polysorbate-80 (P80) does not result in an aggravated phenotype, suggesting they act through similar mechanisms to induce chronic intestinal inflammation and metabolic dysregulations. Indeed, and despite their distinct effects on microbiota composition, both CMC and P80 promote microbiota encroachment, contributing to chronic intestinal inflammation and metabolic dysregulations, likely through immune activation and pro-inflammatory gene expression.^16,25^

The doses of emulsifiers used in this study are relatively high compared to the averaged human exposure. Of note, the NutriNet-Santé cohort provides important epidemiological data linking human consumption of various emulsifiers to health outcomes,^26–28^ and we aimed here to mimic exposure to numerous emulsifiers consumed on a daily basis, thus justifying the relatively high dose used. In the future, studies should test, in mouse models, the effect of emulsifier cocktails at doses mimicking human consumption.

To directly assess the role played by microbiota encroachment and the mucus-associated microbial ecosystem in driving chronic low-grade intestinal inflammation and metabolic dysregulation, we transplanted mucus-associated microbiota from emulsifier-treated mice into germ-free naive mice. This approach revealed that a dysregulated mucus-associated microbial ecosystem, driven by dietary emulsifier consumption, especially CMC, is sufficient to transfer microbiota encroachment and replicate low-grade intestinal inflammation and metabolic dysregulations in recipient mice. This supports the notion that mucus-associated bacteria can solely and directly regulate intestinal inflammatory tone as well as host metabolism.

16S rRNA sequencing analysis revealed that all genera present in the recipient mice’s microbiota were present in the donor mice; while only 34% of the species from the donor’s mucus microbiota were observed to be present in the recipient mice. This suggests that while our Isocage-based approach efficiently prevented recipient mice from environmental contamination, we only partially transplanted the donor’s mucus microbiota. This suggests that either only select mucus-associated microbiota members, efficiently transplanted here, are sufficient to drive chronic low-grade intestinal inflammation and metabolic dysregulations, or that the recipient mice could have experienced more severe chronic inflammation and metabolic dysregulations following a more efficient microbiota transfer.

Consumption of dietary emulsifiers depletes select microbiota features, including the *Alloprevotella* genus, which is typically associated with the production of short-chain fatty acids (SCFAs) through the fermentation of dietary fibers.^29–31^ SCFAs have beneficial effects, including anti-inflammatory properties and modulation of metabolism.^32–35^ Higher amounts of *Alloprevotella* correlate with healthier metabolic profiles,^36,37^ likely due to its role in carbohydrate metabolism and fatty acid synthesis.^38^ Hence, the depletion of *Alloprevotella* in emulsifier-treated mice might contribute to an imbalanced intestinal microbial populations, leading to increased gut permeability, inflammation, and metabolic endotoxemia – all factors implicated in the development of metabolic diseases.^39–42^

We previously reported that emulsifier-treated mice exhibit a depletion in colonic cells and an increase in mucin-degrading microbiota members, both potentially contributing to the impaired colonic mucus layer and associated microbiota encroachment.^11,25^ In the current study, we observed that mucus-associated microbiota transplantation can efficiently transfer microbiota encroachment phenomena, suggesting that this bacterial population includes mucus degraders and/or bacteria able to directly downregulate colonic mucus expression. Although this study was performed with male mice, previous research suggests that emulsifier-induced encroachment and metabolic dysregulation is consistent in both male and female mice, suggesting a sex-independent mechanism.^11^

To conclude, we report that mucus-associated bacteria hold the potential to solely and directly drive chronic low-grade intestinal inflammation and downstream metabolic dysfunctions. While further investigations appear needed to identify mucus-associated bacterial species and strains involved in driving the inflammatory response, as well as to characterize mechanism by which select mucus-associated microbiota members encroach into the mucus layer and trigger intestinal inflammation, our findings nonetheless open the opportunity to develop microbiome-targeted approaches aimed at modulating this inner mucus-associated population. Such approaches would for example aim at modulating this mucus-associated microbial niche in a way that confers protection against intestinal inflammation and associated diseases. Indeed, we previously reported that modulation of the host–microbiota interaction at the mucosal side offers efficient protection against microbiota-mediated diseases.^25,43^ Future work should decipher which mucus-associated microbiota members are impacted by dietary emulsifier consumption, aiming to develop strategies to prevent/counteract microbiota encroachment and, consequently, downstream chronic inflammation.

## Methods

### Mice and reagents

Mice were either housed at Georgia State University, Atlanta, Georgia, USA under institutionally-approved protocols (IACUC # A14033 for Figure 3) or at the Institut Cochin, Paris, France (APAFIS#247882019102806256593 v8 for Figures 1, 2, 4, S1, S2, and S3). Mice were fed Purina rodent chow #5001 when concomitantly treated with dietary emulsifiers, or Purina rodent autoclavable chow #5021 when germfree mice were colonized with mucus-associated microbiota members. Sodium carboxymethylcellulose (CMC, Cat # 419311 average MW ~ 250,000) and Polysorbate-80 (P80, Cat # P1754) were purchased from Sigma (Sigma, St. Louis, MO).

### Treatment with dietary emulsifiers

Wild-type C57BL/6 J male mice were purchased from Janvier and exposed to regular drinking water (control group, *N* = 15), CMC (1% w/v, *N* = 15), P80 (1% v/v, *N* = 15) or a combination of CMC and P80 (1% w/v and 1% v/v, respectively, *N* = 15) diluted in the drinking water for 16 weeks, with solutions changed every week. Body weights were measured every week and fresh feces were collected every week for downstream analysis. After 4 months of emulsifiers treatment, mice were fasted for 15-h at which time blood was collected through the retrobulbar intra-orbital capillary plexus. Hemolysis-free serum was generated by centrifugation of blood using serum separator tubes (Becton Dickinson, Franklin Lakes, NJ). Mice were then euthanized, and colon length, colon weight, spleen weight, and peri-epididymal fat pad deposition weight were measured. Organs were collected for downstream analysis. Mucosal biopsy homogenates were suspended in 30% glycerol in PBS and stocked at − 80°C until further analysis.

### Overnight blood glucose measurement

Mice were placed in a clean cage and fasted (removal of food) for 15 h. Blood glucose concentration was then determined using a Nova Max Plus Glucose Meter and expressed in mg/dL.

### Germ-free mice-based experiments and mucus-associated bacteria transplantation

Wild-type, Germ-free C57BL/6 male mice were purchased from CDTA Orléans and transported under germ-free conditions to our gnotobiotic facility at the Institut Cochin. Upon arrival, mice were orally administered 200 μL of biopsy homogenates suspension collected from either water-treated (*N* = 9), CMC-treated (*N* = 9) or P80-treated (*N* = 8) mice. The transfer of mucus-associated microbiota was performed by combining homogenized colonic biopsies from mice of each experimental group (water-, CMC- or P80-treated groups) and transplanting such microbial suspension by oral gavage (200 µl of the mixed homogenates) to each recipient germ-free mice. Transplanted mice were then fed Purina rodent autoclavable chow #5021 and monitored as previously described.^11^ Six weeks post-transplantation, mice were then challenged with a high-fat diet for additional 6 weeks. After this HFD regimen, mice were fasted for 15-h for overnight fasting blood glucose measurement and then euthanized the following day. Colon length, colon weight, spleen weight and peri-epididymal fat pad deposition weight were measured. Organs and serum were collected for downstream analysis.

### H&E staining and histopathologic analysis

Following euthanasia, mouse colons and small intestines were fixed in Carnoy solution and then embedded in paraffin. Tissues were sectioned at 5-μm thickness and stained with hematoxylin & eosin (H&E) using standard protocols. H&E-stained slides were scored as follows: each colon was assigned four scores based on the degree of epithelial damage and inflammatory infiltrate in the mucosa, submucosa and muscularis/serosa, as previously described.^44,45^ The 4 individual scores per colon were added, resulting in a total scoring range of 0–12 per mouse.

### Liver steatosis quantification

Liver steatosis was quantified by analyzing hepatocellular vesicular steatosis, i.e., macrovesicular steatosis and microvesicular steatosis separately, as well as by hepatocellular hypertrophy quantification, as previously described.^46^ Inflammation was scored by analyzing the amount of inflammatory cell aggregates. Macrovesicular steatosis and microvesicular steatosis were separately scored and the severity was graded, based on the percentage of the total area affected, into the following categories: 0 (0–5%), 1 (5–33%), 2 (34–66%) and 3 (66–100%). The difference between macrovesicular and microvesicular steatosis was defined by whether the vacuoles displaced the nucleus to the side (macrovesicular) or not (microvesicular). Similarly, the level of hepatocellular hypertrophy, defined as cellular enlargement more than 1.5 times the normal hepatocyte diameter, was scored, based on the percentage of the total area affected, into the following categories: 0 (0–5%), 1 (5–33%), 2 (34–66%) and 3 (66–100%). For hepatocellular hypertrophy, the evaluation was merely based on abnormal enlargement of the cells, irrespective of rounding of the cells and/or changes in cytoplasm or the number of vacuoles, and is therefore not a substitute of ballooning. The unweight sum of the scores for steatosis (macrovesicular steatosis, microvesicular steatosis and hypertrophy) thus ranged from 0 to 9. Both steatosis and hypertrophy were evaluated at a 40 magnification, and terminal hepatic venules and portal tracts were excluded in order to only take hepatocytes into account.

### Immunostaining of mucins and localization of bacteria by FISH

Mucus immunostaining was paired with fluorescent in situ hybridization (FISH), as previously described,^47^ in order to analyze bacteria localization at the surface of the intestinal mucosa.^11,48^ Briefly, colonic tissues (proximal colon, 2nd cm from the cecum) containing fecal material were placed in methanol-Carnoy’s fixative solution (60% methanol, 30% chloroform, 10% glacial acetic acid). Tissues were then washed in methanol 2 × 30 min, ethanol 2 × 15 min, ethanol/xylene (1:1) 15 min and xylene 2 × 15 min, followed by embedding in Paraffin with a vertical orientation. Five μm sections were performed and dewaxed by preheating at 60°C for 10 min, followed by xylene 60°C for 10 min, xylene for 10 min and 99.5% ethanol for 10 min. Hybridization step was performed at 50° C overnight with EUB338 probe (5’- GCTGCCTCCCGTAGGAGT-3’, with a 5’ labeling using Alexa 647) diluted to a final concentration of 10 μg/mL in hybridization buffer (20 mm Tris – HCl, pH 7.4, 0.9 M NaCl, 0.1% SDS, 20% formamide). After washing 10 min in wash buffer (20 mm Tris – HCl, pH 7.4, 0.9 M NaCl) and 3 × 10 min in PBS, PAP pen (Sigma, St. Louis, MO) was used to mark around the section and block solution (5% fetal bovine serum in PBS) was added for 30 min at 4°C. Mucin-2 primary antibody (rabbit H-300, Santa Cruz Biotechnology) was diluted 1:1500 in block solution and apply overnight at 4°C. After washing 3 × 10 min in PBS, block solution containing anti-rabbit Alexa 488 secondary antibody diluted 1:1500, Phalloidin-Tetramethylrhodamine B isothiocyanate (Sigma, St. Louis, MO) at 1 μg/mL and Hoechst 33,258 (Sigma, St. Louis, MO) at 10 μg/mL was applied to the section for 2 h. After washing 3 × 10 min in PBS slides were mounted using Prolong anti-fade mounting media (Life Technologies). Observations were performed with a Zeiss LSM 700 confocal microscope with software Zen 2011 version 7.1. This software was used to determine the distance between bacteria and epithelial cell monolayer. For quantifications, 15 bacteria were analyzed per representative section, and 5 representative sections were assessed per mouse. The average distance was then calculated from the 75 measured bacterium-epithelium distances.

### Fecal flagellin and lipopolysaccharide load quantification

We quantified flagellin and lipopolysaccharide (LPS) as previously described^49^ using human embryonic kidney (HEK)-Blue-mTLR5 and HEK-BluemTLR4 cells, respectively (Invivogen, San Diego, California, USA). We resuspended fecal material in PBS to a final concentration of 100 mg/mL and homogenized for 10 s using a Mini-Beadbeater-24 without the addition of beads to avoid bacteria disruption. We then centrifuged the samples at 8000 g for 2 min and serially diluted the resulting supernatant and applied to mammalian cells. Purified *E. coli* flagellin and LPS (Sigma, St Louis, Missouri, USA) were used for standard curve determination using HEK-Blue-mTLR5 and HEK-Blue-mTLR4 cells, respectively. After 24 h of stimulation, we applied cell culture supernatant to QUANTI-Blue medium (Invivogen, San Diego, California, USA) and measured alkaline phosphatase activity at 620 nm after 30 min.

### Fecal flagellin and LPS-specific immunoglobulins

Flagellin- and LPS-specific IgA and IgG levels were quantified by ELISA, as previously reported.^50^ Microtiter plates were coated overnight with purified *E. coli* flagellin (100 ng/well) or LPS (1 μg/well). Fecal samples diluted 1:500 was then applied. After incubation and washing, wells were incubated either with anti-mouse IgA or anti-mouse IgG. Quantification was performed using the colorimetric peroxidase substrate tetramethylbenzidine. Data are reported as OD corrected by subtracting background (determined by the readings obtained in wells not subjected to serum application).

### Inner mucus layer microdissection

Microdissection was performed on an Arcturus® Laser Capture Microdissection system, as previously described.^17^ Inner mucus layers were selected and collected on CapSureTM Macro LCM Caps (Arcturus) using a combination of infrared (IR) capture and ultraviolet (UV) laser cutting. The membrane covering caps were subsequently carefully collected, placed in clean DNA-free 0.5 mL tubes, and store in −80°C until DNA extraction.

### DNA extraction from inner mucus layer-microdissected samples

Qiagen QIAamp DNA Micro Kit was used to isolate DNA from laser-microdissected inner mucus layer. Briefly, 30 μL of buffer ATL and 20 μL of proteinase K were added to the microdissected samples and incubated at 56°C overnight. 50 μL of buffer ATL and 100 μL of buffer AL were added, samples were mixed by vortexing, and 100 μL of ethanol was added followed by a 5 min incubation at room temperature. The sample was next transferred to a QIAamp MinElute column without the membrane and centrifuge at 6,000 g for 1 min. The column was washed with 500 μL of buffer AW1 and 500 μL of buffer AW2 and dried with a 3 min centrifugation at 20,000 g. 20 μL of DNA-free water (Mobio) were then applied to the center of the column, incubated at room temperature for 10 min, followed by a final centrifugation at 20,000 g for 1 min in order to collect eluted DNA.

### DNA extraction from fecal samples

Total bacterial DNA was isolated from weighted feces using the DNeasy 96 PowerSoil Pro QIAcube HT kit with mechanical disruption (bead-beating) and the QIAcube HT instrument paired to the QIAcube HT Prep Manager software (Qiagen).

### Microbiota analysis by 16S rRNA gene sequencing using illumina technology

16S rRNA gene amplification and sequencing were done using the Illumina MiSeq technology following the protocol of Earth Microbiome Project with their modifications to the MOBIO PowerSoil DNA Isolation Kit procedure for extracting DNA (www.earthmicrobiome. org/emp-standard-protocols). Bulk DNA were extracted from frozen extruded feces using a DNeasy 96 PowerSoil Pro QIAcube HT kit from Qiagen (Hilden, Germany) with mechanical disruption (bead-beating). The 16S rRNA genes, region V4, were PCR amplified from each sample using a composite forward primer and a reverse primer containing a unique 12-base barcode, designed using the Golay error-correcting scheme, which was used to tag PCR products from respective samples.^51^ We used the forward primer 515F 5’- *AATGATACGGCGACCACCGAGATCTACACGCT*XXXXXXXXXXXX**TATGGTAATT*GT***GTGYCAGCMGCCGCGGTAA-3’: the italicized sequence is the 5’ Illumina adapter, the 12 X sequence is the golay barcode, the bold sequence is the primer pad, the italicized and bold sequence is the primer linker and the underlined sequence is the conserved bacterial primer 515F. The reverse primer 806 R used was 5’-*CAAGCAGAAGACGGCATACGAGAT***AGTCAGCCAG*CC*** GGACTACNVGGGTWTCTAAT-3’: the italicized sequence is the 3’ reverse complement sequence of Illumina adapter, the bold sequence is the primer pad, the italicized and bold sequence is the primer linker and the underlined sequence is the conserved bacterial primer 806 R. PCR reactions consisted of Hot Master PCR mix (Quantabio, Beverly, MA, USA), 0.2 μM of each primer, 10–100 ng template, and reaction conditions were 3 min at 95°C, followed by 30 cycles of 45 s at 95°C, 60 s at 50°C, and 90 s at 72°C on a Biorad thermocycler. Products were then visualized by gel electrophoresis and quantified using Quant-iT PicoGreen dsDNA assay (Clariostar Fluorescence Spectrophotometer). A master DNA pool was generated in equimolar ratios, subsequently purified with AmPure magnetic purification beads (Agencourt, Brea, CA, USA) and sequenced using an Illumina MiSeq sequencer (paired-end reads, 2 × 250 bp) at the Genom’IC platform (INSERM U1016, Paris, France).

### 16S rRNA gene sequence analysis

16S rRNA sequences were analyzed using QIIME2—version 2022.2.^52^ Sequences were demultiplexed and quality filtered using the Dada2 method^53^ with QIIME2 default parameters in order to detect and correct Illumina amplicon sequence data, and a table of Qiime 2 artifact was generated. A tree was next generated for phylogenetic diversity analyses, and alpha and beta diversity analyses were computed using the core-metrics-phylogenetic command. PCoA plots were used to assess the variation between the experimental group (beta diversity). For taxonomy analysis, features were assigned to operational taxonomic units (OTUs) with a 99% threshold of pairwise identity to the Greengenes reference database 2022_10.^54^ Data Availability: Unprocessed sequencing data are deposited in the European Nucleotide Archive under accession number PRJEB83801, publicly accessible at https://www.ebi.ac.uk/ena/browser/home.

### Data presentation and statistical analysis

Data are expressed as means ± SD and statistical analyses were performed using GraphPad Prism software (V.8.2.0). Significance was determined using one-way analysis of variance (ANOVA), followed by a Dunnett post hoc test and significant differences were noted as follows: *p ≤ 0.05 **p ≤ 0.01 ***p ≤ 0.001 ****p ≤ 0.0001. For clustering analyzing on principal coordinate plots, categories were compared, and statistical significance of clustering were determined via Permanova. For statistical analysis of microbiota data, the most significantly differentially abundant features were identified using Microbiome Multivariable Associations with Linear Models (MaAsLin 2).^55^ Threshold for Heatmaps was set at q < 0.05.

## Supplementary Material

Supplemental Material

## Abbreviations

ANOVA: Analysis of variance
ASV: Amplicon sequence variant
CMC: Carboxymethylcellulose
FISH: Fluorescent *in situ* Hybridization
FliC: Flagellin
HFD: High Fat Diet
IBD: Inflammatory Bowel Disease
Ig: Immunoglobulins
Lcn2: Lipocalin 2
LPS: Lipopolysaccharide
MaAsLin2: Microbiome Multivariable Associations with Linear Models
MAMPs: Microbe-Associated Molecular Patterns
PCoA: Principal Coordinate Analysis
P80: Polysorbate 80
SD: Standard deviation
