# Supplementary material for: Mucus-penetrating microbiota drive chronic low-grade intestinal inflammation and metabolic dysregulation
Source: Gut Microbes. 2025 Jan 26;17(1):2455790. doi: 10.1080/19490976.2025.2455790 (PMC11776472; doi:10.1080/19490976.2025.2455790)
Supplement: Supplemental Material [file KGMI_A_2455790_SM9144.docx]

**Supplemental figure legends**

**Figure S1. Dietary emulsifiers consumption reproducibly drives intestinal microbiota alterations.** Mice were given drinking water (blue) containing 1.0% CMC (orange), P80 (purple), or a combination of both (grey). (**A**) Mice were weighed weekly for 16 weeks. At the end of the study, fecal DNA was extracted and analyzed through 16S rRNA gene amplification and Illumina sequencing. (**B)** The Simpson diversity index was computed, and (**C**) a taxonomical bar plot representation of the relative abundances of the various taxa present at the family level was generated. (**D**) Using Microbiome Multivariable Associations with Linear Models (MaAslin 2), the significantly differentially abundant features were identified and presented in a heatmap.

**Figure S2. Germ-free mice transplanted with mucus-associated microbiota exhibit group-based microbiota clustering.** Germ-Free mice were transplanted with mucosal biopsy homogenates collected from mice treated with water only (blue), CMC (orange) or P80 (purple). Bacterial DNA was extracted from feces collected longitudinally and subjected to 16S rRNA gene amplification and Illumina-based sequencing. (**A-F**) Data are presented as Principal Coordinate Analysis (PCoA) of the unweighted Unifrac distance matrix. Each dot represents an individual animal and is color-coded (blue: water, orange: CMC, purple: P80).

**Figure S3. Germ-free mice transplanted with mucus-associated microbiota exhibit alterations in their microbiota composition and localization.** Germ-Free mice were transplanted with mucosal biopsy homogenates collected from mice treated with water only (blue), CMC (orange) or P80 (purple). Bacterial DNA was extracted from feces longitudinally collected and subjected to 16S rRNA gene amplification and Illumina-based sequencing. (**A**) A taxonomical bar plot representation of the relative abundances of the various taxa present at the family level was generated. (**B**) Significantly differentially abundant features were identified at week 12 using Microbiome Multivariable Associations with Linear Models (MaAslin 2) and displayed as heatmaps. (**C**) Longitudinal analysis of the Simpson diversity index of the intestinal microbiota assessed by 16S rRNA gene sequencing was calculated. Simpson indices were normalized to water, defined as 1. (**D**) Representative pictures obtained from 5 biological replicates. MUC2, green; actin, purple; bacteria, red; and DNA, blue. Scale bar, 50 µm

220

**A**

200

Body weight gain (%)

180

160

140

120

100


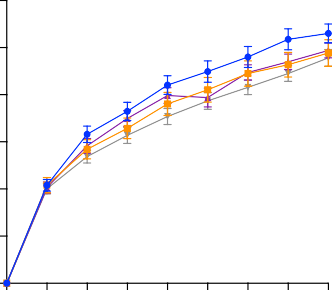


0 2 4 6 8 10 12 14 16

Water CMC P80


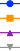
CMC+P80

ns

0.99


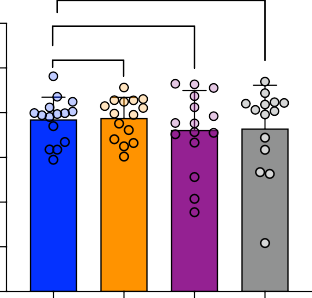


ns

ns

**B**

0.98

0.97

Simpson index

0.96

0.95

0.94

0.93

Water

CMC

P80

CMC+P80

100

**C**

80

Relative abundances (%)

60

40

20

0


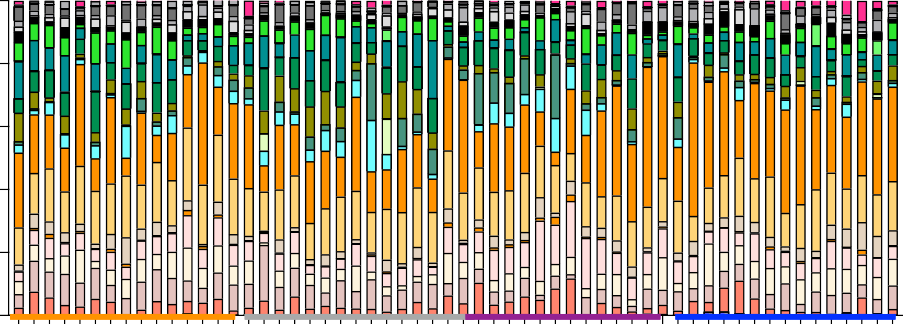


## CMC CMC_P80 P80 WATER


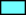
k Bacteria;p Cyanobacteria;c 4C0d-2;o YS2;f


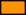

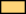
k Bacteria;p Bacteroidetes;c Bacteroidia;o Bacteroidales;f Rikenellaceae k Bacteria;p Bacteroidetes;c Bacteroidia;o Bacteroidales;f S24-7


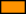
k Bacteria;p Bacteroidetes;c Bacteroidia;o Bacteroidales;f Porphyromonadaceae


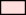
k Bacteria;p Bacteroidetes;c Bacteroidia;o Bacteroidales;f Bacteroidaceae


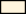

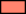
k Bacteria;p Bacteroidetes;c Bacteroidia;o Bacteroidales;f [Paraprevotellaceae] k Bacteria;p Bacteroidetes;c Bacteroidia;o Bacteroidales;f


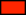
k Bacteria;p Bacteroidetes;c Bacteroidia;o Bacteroidales;


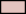

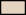
k Bacteria;p Bacteroidetes;c Bacteroidia;o Bacteroidales;f [Odoribacteraceae] k Bacteria;p Bacteroidetes;c Bacteroidia;o Bacteroidales;f Prevotellaceae


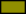
k Bacteria;p Firmicutes;c Clostridia;o Clostridiales;


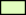
k Bacteria;p Firmicutes;c Bacilli;o Lactobacillales;f Lactobacillaceae


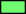
k Bacteria;p Firmicutes;c Clostridia;o Clostridiales;f Ruminococcaceae


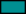

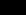
k Bacteria;p Firmicutes;c Erysipelotrichi;o Erysipelotrichales;f Erysipelotrichaceae k Bacteria;p Firmicutes;c Clostridia;o Clostridiales;f Lachnospiraceae


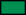
k Bacteria;p Firmicutes;c Clostridia;o Clostridiales;f


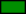

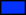
k Bacteria;p Firmicutes;c Clostridia;o Clostridiales;f Dehalobacteriaceae k Bacteria;p Firmicutes;c Bacilli;o Lactobacillales;f Streptococcaceae


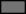

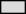
k Bacteria;p Proteobacteria;c Deltaproteobacteria;o Desulfovibrionales;f Desulfovibrionaceae k Bacteria;p Proteobacteria;c Alphaproteobacteria;o RF32;f


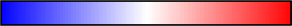

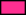
k Bacteria;p Verrucomicrobia;c Verrucomicrobiae;o Verrucomicrobiales;f Verrucomicrobiaceae


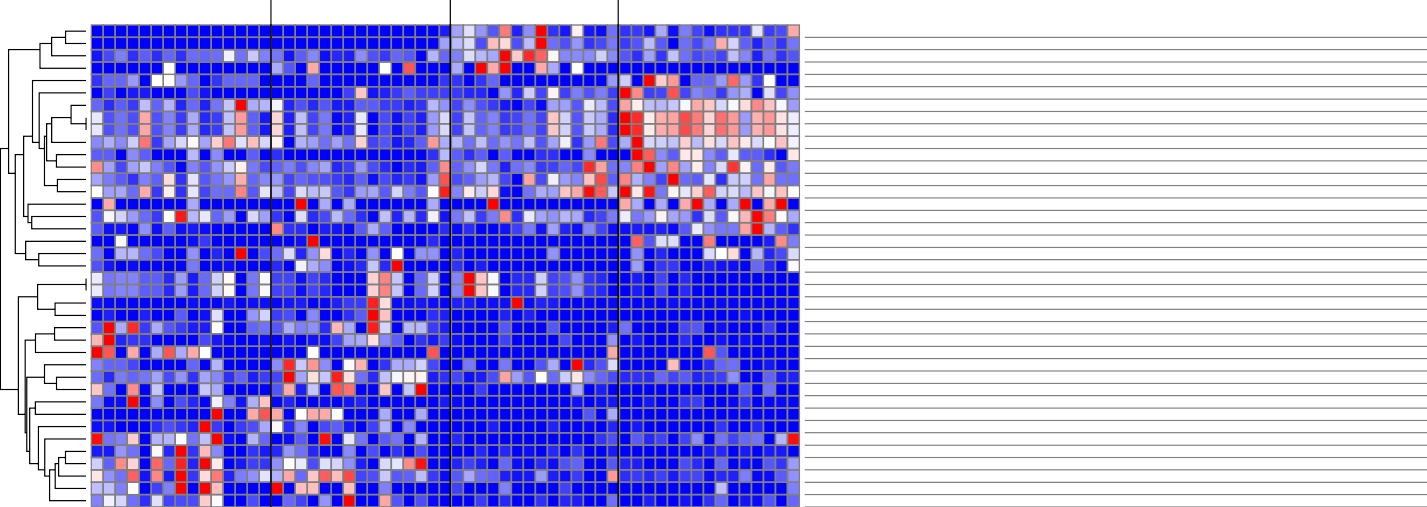


**D**

**CMC**

**CMC_P80**

**P80**

**Water**

row min

row max

d Bacteria;p Firmicutes_A;c Clostridia_258483;o Oscillospirales;f Ruminococcaceae;g UBA946

d Bacteria;p Firmicutes_A;c Clostridia_258483;o Oscillospirales;f Ruminococcaceae;g Ruminococcus_C_58660 d Bacteria;p Bacteroidota;c Bacteroidia;o Bacteroidales;f Bacteroidaceae;g Bacteroides_H

d Bacteria;p Firmicutes_D;c Bacilli;o RF39;f UBA660;g Faecimonas

d Bacteria;p Firmicutes_A;c Clostridia_258483;o Christensenellales;f CAG-74;

d Bacteria;p Bacteroidota;c Bacteroidia;o Bacteroidales;f Muribaculaceae;g Paramuribaculum d Bacteria;p Bacteroidota;c Bacteroidia;o Bacteroidales;f Muribaculaceae;

d Bacteria;p Bacteroidota;c Bacteroidia;o Bacteroidales;f Muribaculaceae;g UBA3263 d Bacteria;p Bacteroidota;c Bacteroidia;o Bacteroidales;f Muribaculaceae;g UBA3263

d Bacteria;p Bacteroidota;c Bacteroidia;o Bacteroidales;f Bacteroidaceae;g Alloprevotella d Bacteria;p Firmicutes_A;c Clostridia_258483;o Oscillospirales;f UBA644;g UBA644

d Bacteria;p Firmicutes_D;c Bacilli;o Erysipelotrichales;f Erysipelotrichaceae;g Dubosiella

d Bacteria;p Proteobacteria;c Gammaproteobacteria;o Burkholderiales_595427;f Burkholderiaceae_A_595427;g Turicimonas d Bacteria;p Bacteroidota;c Bacteroidia;o Bacteroidales;f Muribaculaceae;g UBA7173

d Bacteria;p Firmicutes_A;c Clostridia_258483;o Oscillospirales;f Acutalibacteraceae;g Eubacterium_R d Bacteria;p Bacteroidota;c Bacteroidia;o Bacteroidales;f Bacteroidaceae;g Prevotella

d Bacteria;p Verrucomicrobiota;c Verrucomicrobiae;o Verrucomicrobiales;f Akkermansiaceae;g Akkermansia d Bacteria;p Firmicutes_A;c Clostridia_258483;o Lachnospirales;f Lachnospiraceae;g Robinsoniella

d Bacteria;p Firmicutes_A;c Clostridia_258483;o Oscillospirales;f CAG-272;g Avispirillum

d Bacteria;p Firmicutes_A;c Clostridia_258483;o Lachnospirales;f Lachnospiraceae;g CAG-95 d Bacteria;p Proteobacteria;c Alphaproteobacteria;o Rs-D84_512864;f Rs-D84;g Enterousia

d Bacteria;p Proteobacteria;c Alphaproteobacteria;o Rs-D84_512864;f Rs-D84;g Enterousia d Bacteria;p Firmicutes_D;c Bacilli;o Lactobacillales;f Lactobacillaceae;g Ligilactobacillus

d Bacteria;p Firmicutes_D;c Bacilli;o Erysipelotrichales;f Coprobacillaceae;g Erysipelatoclostridium

d Bacteria;p Firmicutes_A;c Clostridia_258483;o Oscillospirales;f Ruminococcaceae;g Massilioclostridium d Bacteria;p Firmicutes_A;c Clostridia_258483;o Lachnospirales;f Lachnospiraceae;g Ventrimonas

d Bacteria;p Firmicutes_A;c Clostridia_258483;o Lachnospirales;f Lachnospiraceae;g Acetitomaculum d Bacteria;p Firmicutes_A;c Clostridia_258483;o Lachnospirales;f Lachnospiraceae;g Sporofaciens

d Bacteria;p Firmicutes_A;c Clostridia_258483;o Christensenellales;f CAG-314;g CAG-314

d Bacteria;p Firmicutes_A;c Clostridia_258483;o Lachnospirales;f Lachnospiraceae;g Eubacterium_S d Bacteria;p Firmicutes_D;c Bacilli;o RF39;f UBA660;

d Bacteria;p Firmicutes_D;c Bacilli;o RF39;f UBA660;g CAG-605

d Bacteria;p Firmicutes_A;c Clostridia_258483;o Lachnospirales;f Lachnospiraceae;g Clostridium_AP

d Bacteria;p Firmicutes_A;c Clostridia_258483;o Oscillospirales;f Oscillospiraceae_88309;g Lawsonibacter d Bacteria;p Firmicutes_A;c Clostridia_258483;o Oscillospirales;f Ruminococcaceae;

d Bacteria;p Firmicutes_A;c Clostridia_258483;o Oscillospirales;f Ruminococcaceae;g Phocea d Bacteria;p Cyanobacteria;c Vampirovibrionia;o Gastranaerophilales;f Gastranaerophilaceae;g

d Bacteria;p Firmicutes_A;c Clostridia_258483;o Lachnospirales;f Lachnospiraceae;g Lachnospira

d Bacteria;p Firmicutes_A;c Clostridia_258483;o Oscillospirales;f Oscillospiraceae_88309;g Faecousia

**Figure S1:** Dietary emulsifiers consumption reproducibly drives intestinal microbiota alterations.

**A** *Week 2*


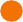

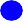

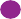

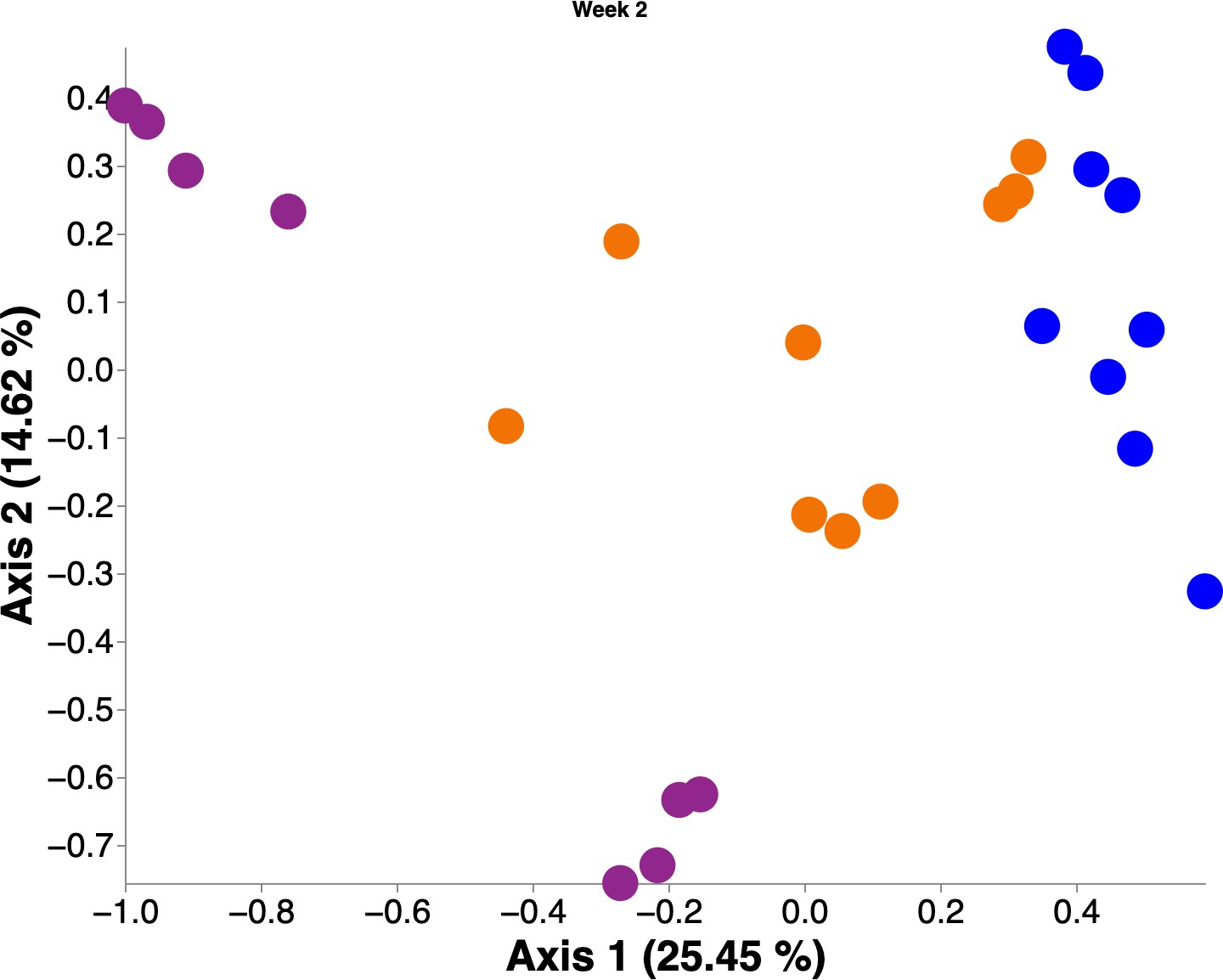


**C** *Week 6*

Water CMC P80

r

Water CMC P80


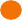

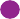

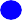

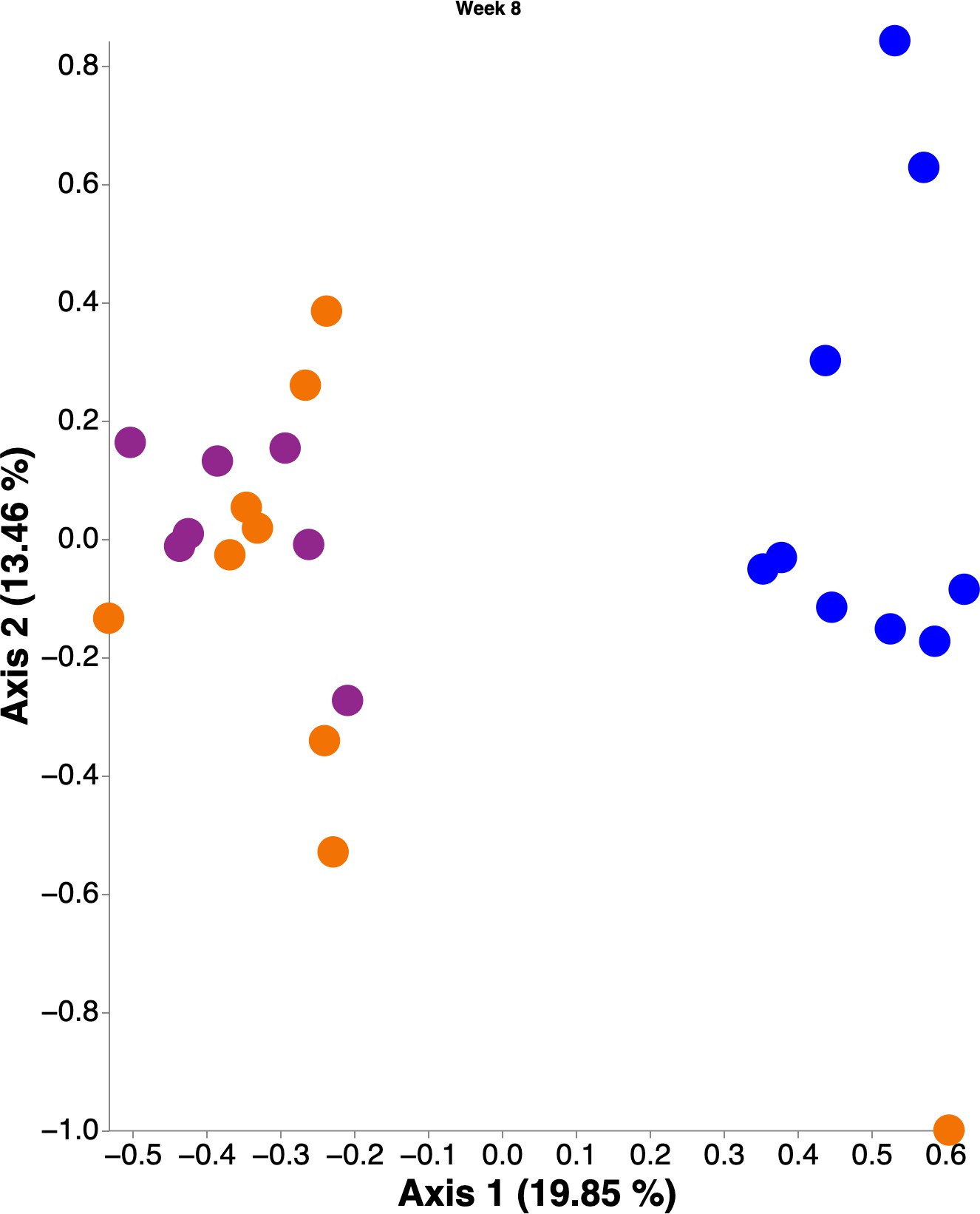

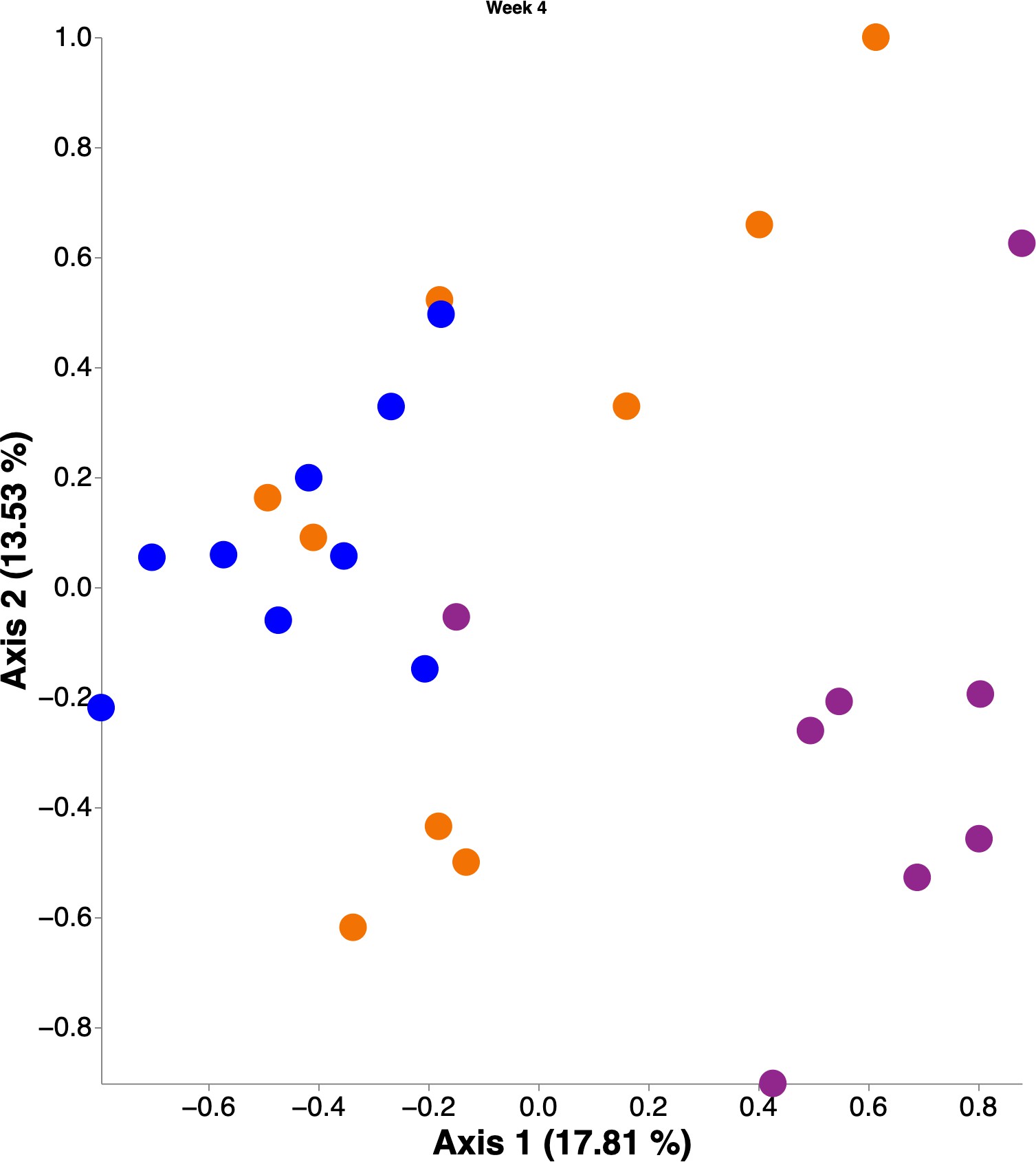

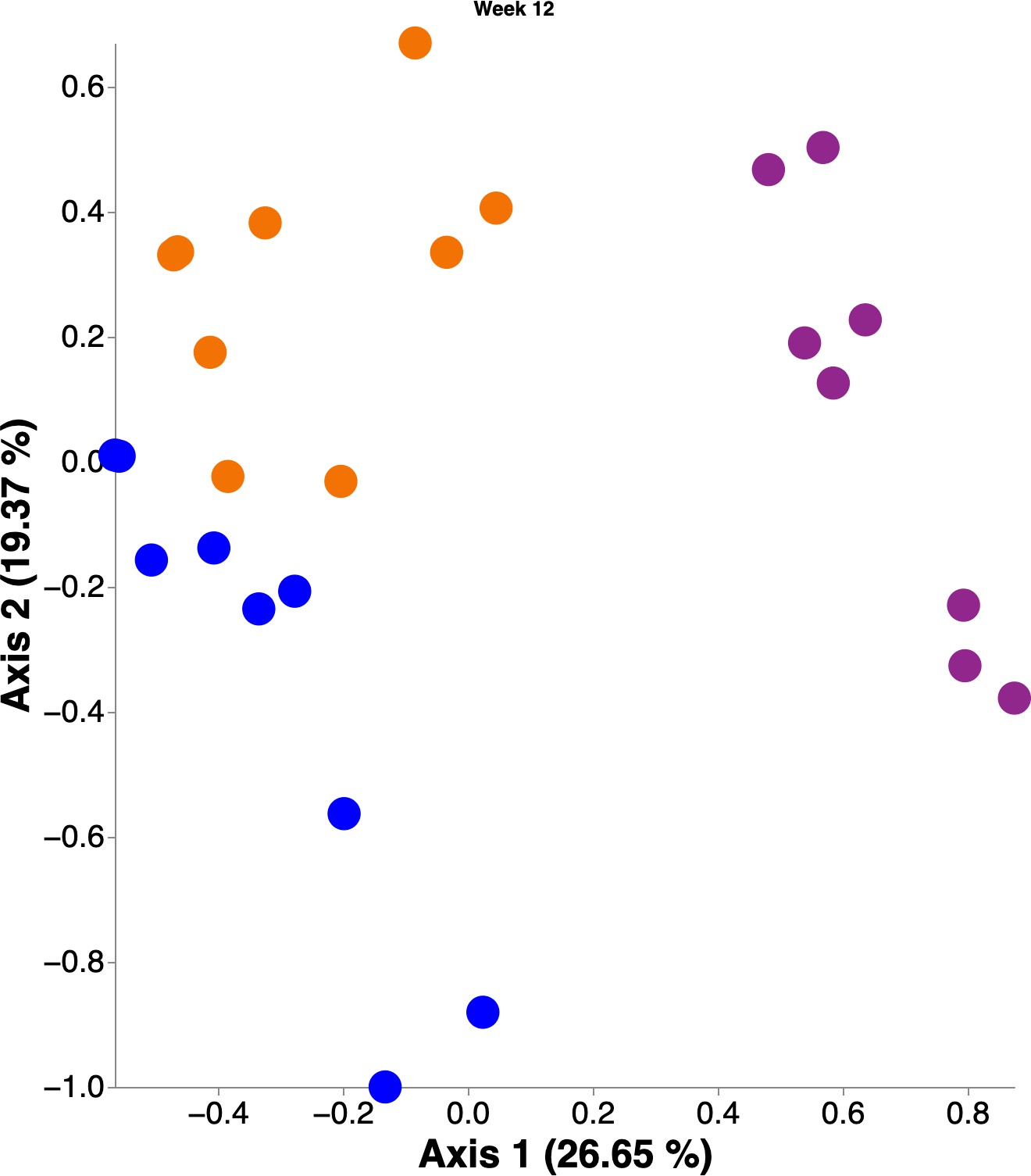

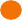

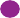

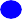


**B**

*Week 4*

Wate CMC

P80

**D**

*Week 8*

Water CMC

P80

*Week 12*

Water CMC

P80


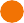

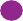

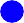

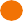

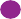

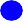


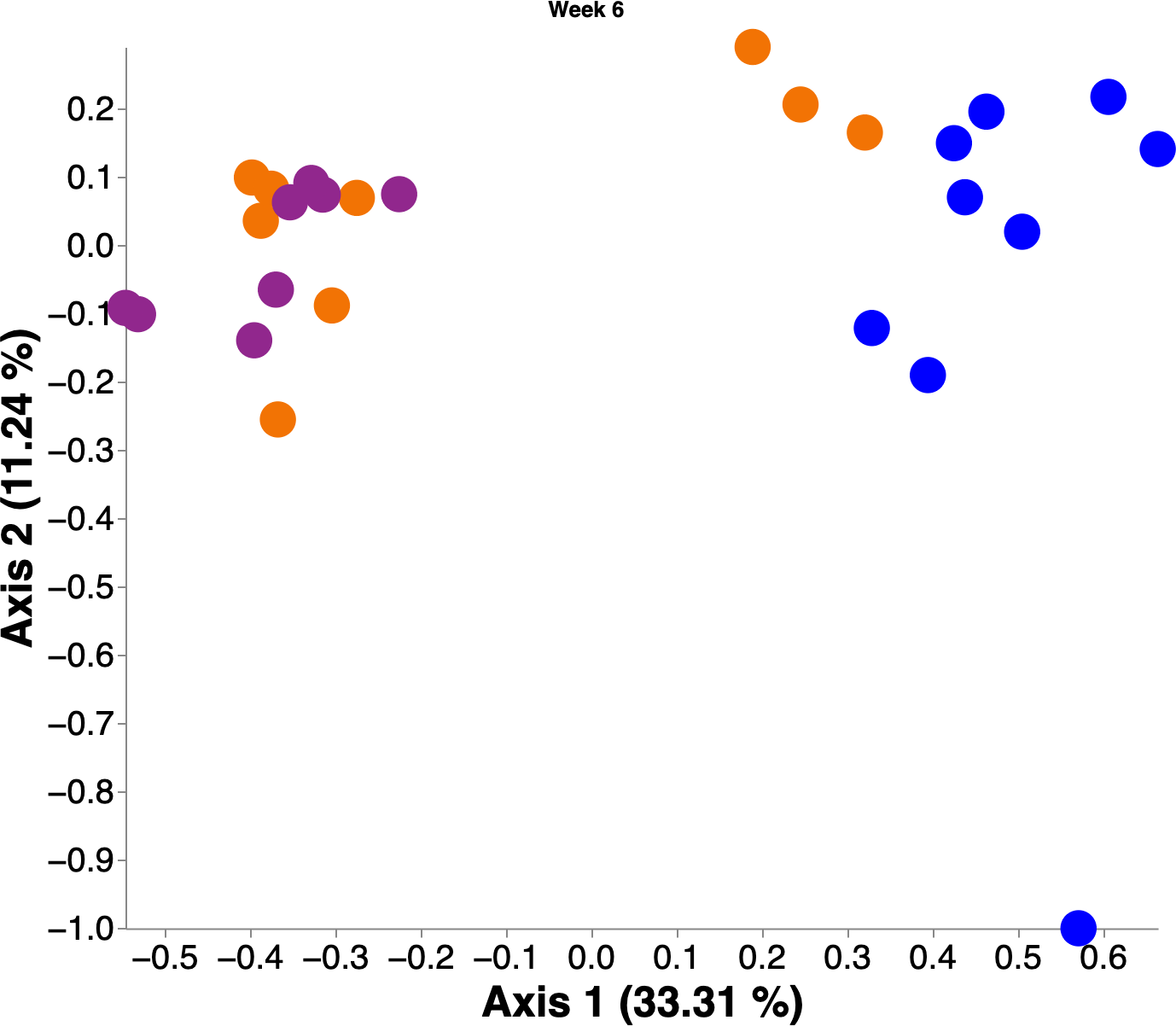


**E** *Week 10*

**F** *Week 12*


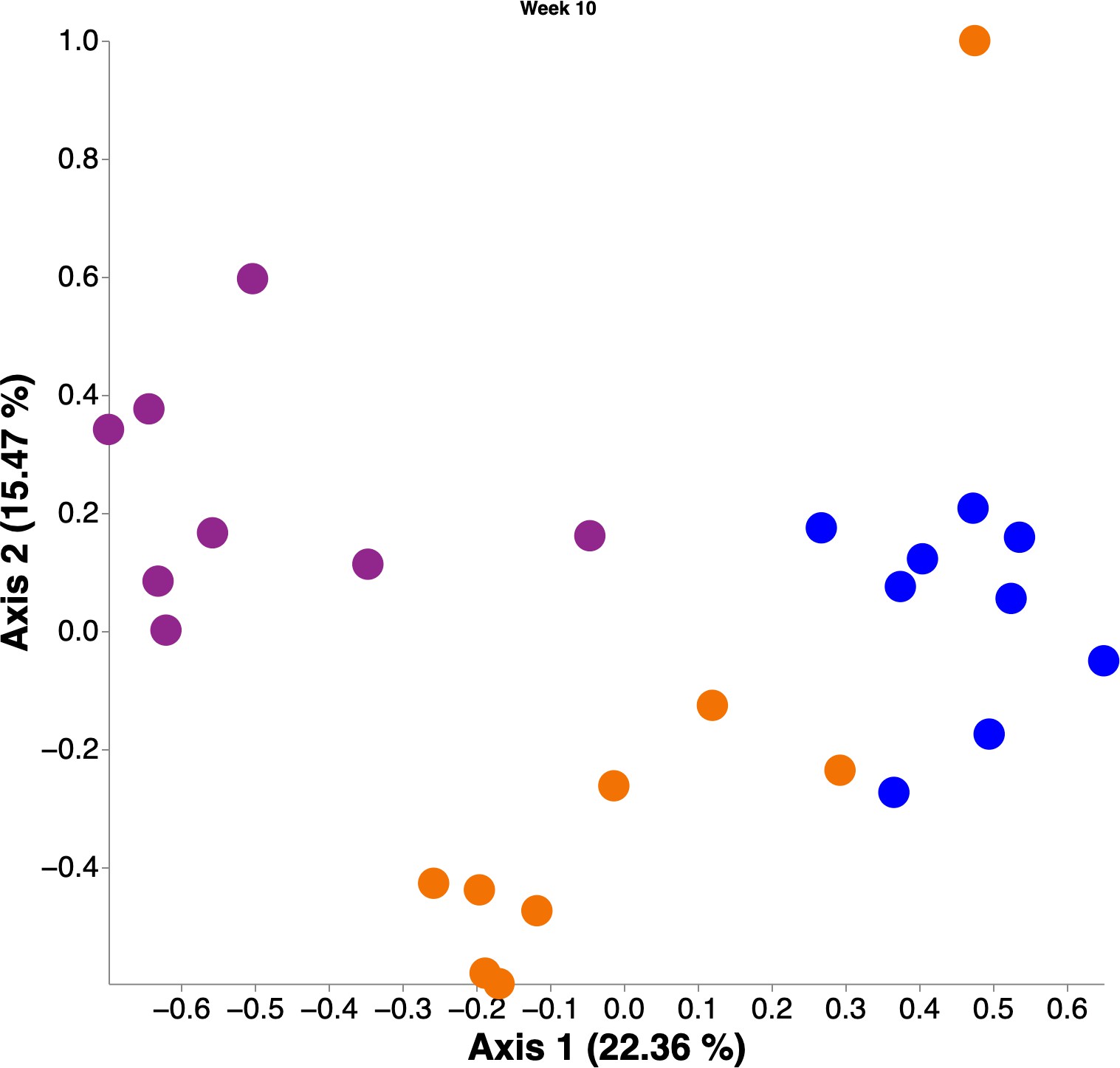

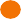

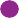

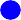


Water CMC

P80

**Figure S2:** Germ-free mice transplanted with mucus-associated microbiota exhibit group-based microbiota clustering.

**A** 100

80

Relative abundance (%)

60

40

20

*Week 12*


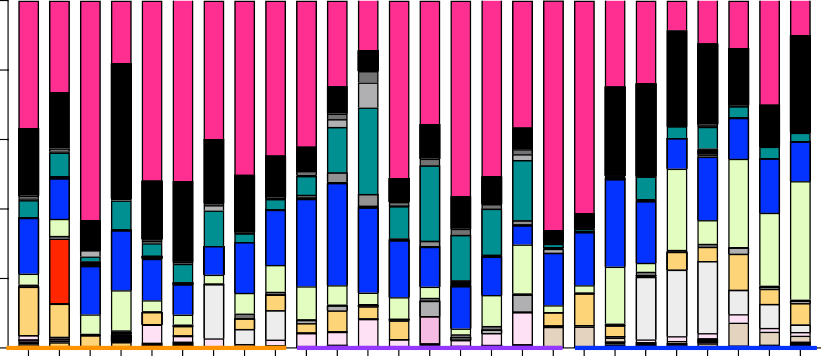
0

CMC

P80 WATER


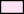
 k Bacteria;p Bacteroidetes;c Bacteroidia;o Bacteroidales;f Bacteroidaceae
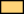
 k Bacteria;p Bacteroidetes;c Bacteroidia;o Bacteroidales;f Rikenellaceae


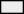
 k Bacteria;p Bacteroidetes;c Bacteroidia;o Bacteroidales;f Porphyromonadaceae
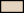
 k Bacteria;p Bacteroidetes;c Bacteroidia;o Bacteroidales;f [Paraprevotellaceae]
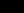
 k Bacteria;p Firmicutes;c Erysipelotrichi;o Erysipelotrichales;f Erysipelotrichaceae
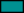
 k Bacteria;p Firmicutes;c Clostridia;o Clostridiales;f Lachnospiraceae


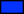
 k Bacteria;p Firmicutes;c Bacilli;o Lactobacillales;f Streptococcaceae


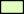
 k Bacteria;p Firmicutes;c Bacilli;o Lactobacillales;f Lactobacillaceae


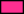
 k Bacteria;p Verrucomicrobia;c Verrucomicrobiae;o Verrucomicrobiales;f Verrucomicrobiaceae

#
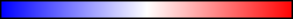
B

row min row max


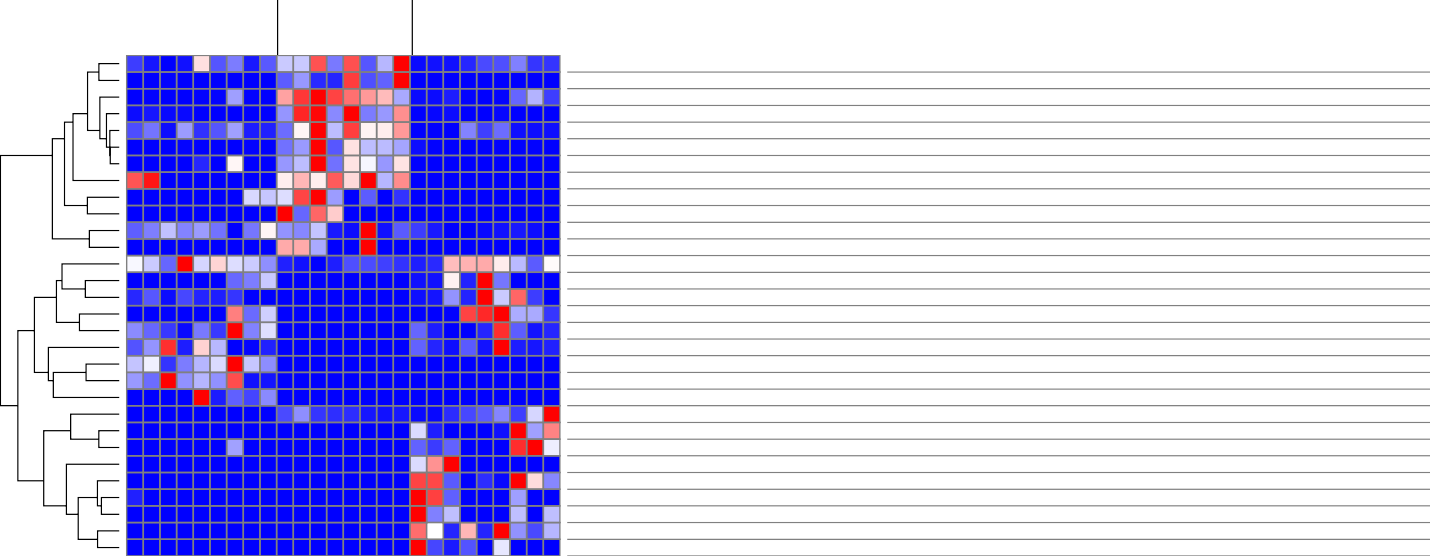


**CMC**

**P80**

**Water**

d Bacteria;p Bacteroidota;c Bacteroidia;o Bacteroidales;f Bacteroidaceae;g Bacteroides_H

d Bacteria;p Bacteroidota;c Bacteroidia;o Bacteroidales;f Muribaculaceae;g CAG-485

d Bacteria;p Firmicutes_D;c Bacilli;o Erysipelotrichales;f Erysipelotrichaceae;g Clostridium_AQ

d Bacteria;p Firmicutes_A;c Clostridia_258483;o Clostridiales;f Clostridiaceae_222000;g Clostridium_T d Bacteria;p Firmicutes_A;c Clostridia_258483;o Lachnospirales;f Lachnospiraceae;g Blautia_A_141780 d Bacteria;p Firmicutes_A;c Clostridia_258483;o Oscillospirales;f Acutalibacteraceae;g Acutalibacter

d Bacteria;p Firmicutes_A;c Clostridia_258483;o Lachnospirales;f Lachnospiraceae;g Anaerostipes

d Bacteria;p Firmicutes_A;c Clostridia_258483;o Oscillospirales;f Oscillospiraceae_88309;g Lawsonibacter d Bacteria;p Firmicutes_D;c Bacilli;o Erysipelotrichales;f Coprobacillaceae;

d Bacteria;p Firmicutes_A;c Clostridia_258483;o Oscillospirales;f Ruminococcaceae;g Angelakisella d Bacteria;p Firmicutes_A;c Clostridia_258483;o Lachnospirales;f Lachnospiraceae;

d Bacteria;p Firmicutes_A;c Clostridia_258483;o Oscillospirales;f Butyricicoccaceae;g Pseudobutyricicoccus d Bacteria;p Firmicutes_D;c Bacilli;o Erysipelotrichales;f Erysipelotrichaceae;g Dubosiella

d Bacteria;p Firmicutes_D;c Bacilli;o Lactobacillales;f Lactobacillaceae;g Lactobacillus

d Bacteria;p Firmicutes_D;c Bacilli;o Lactobacillales;f Lactobacillaceae;g Limosilactobacillus

d Bacteria;p Bacteroidota;c Bacteroidia;o Bacteroidales;f Tannerellaceae;g Parabacteroides_B_862066 d Bacteria;p Firmicutes_A;c Clostridia_258483;o Oscillospirales;f Ruminococcaceae;g Anaerotruncus d Bacteria;p Firmicutes_A;c Clostridia_258483;o Lachnospirales;f Lachnospiraceae;g Kineothrix

d Bacteria;p Firmicutes_A;c Clostridia_258483;o Lachnospirales;f Lachnospiraceae;g Robinsoniella

d Bacteria;p Firmicutes_A;c Clostridia_258483;o Peptostreptococcales;f Peptostreptococcaceae_256921;g Clostridioides_A d Bacteria;p Firmicutes_A;c Clostridia_258483;o Oscillospirales;f Ruminococcaceae;g Massilioclostridium

d Bacteria;p Firmicutes_D;c Bacilli;o Erysipelotrichales;f Erysipelotrichaceae;g Faecalibaculum d Bacteria;p Firmicutes_A;c Clostridia_258483;o Lachnospirales;f Lachnospiraceae;g 14-2

d Bacteria;p Firmicutes_A;c Clostridia_258483;o Lachnospirales;f Lachnospiraceae;g Enterocloster d Bacteria;p Bacteroidota;c Bacteroidia;o Bacteroidales;f Muribaculaceae;g UBA7173

d Bacteria;p Bacteroidota;c Bacteroidia;o Bacteroidales;f Bacteroidaceae;g Alloprevotella

d Bacteria;p Firmicutes_A;c Clostridia_258483;o Oscillospirales;f Oscillospiraceae_88309;g Dysosmobacter d Bacteria;p Firmicutes_A;c Clostridia_258483;o Lachnospirales;f Lachnospiraceae;g Copromonas

d Bacteria;p Firmicutes_A;c Clostridia_258483;o Oscillospirales;f Oscillospiraceae_88309;g

d Bacteria;p Firmicutes_A;c Clostridia_258483;o Lachnospirales;f Lachnospiraceae;g Acetatifactor

# C

Simpson index


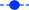
 Water


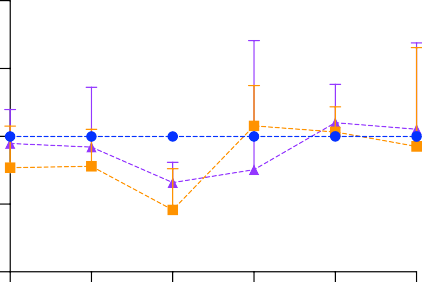


1.4

ns

ns

ns

1.2

ns

ns

1.0

ns

0.8

**

0.6


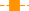
 CMC
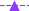
 P80

2 4 6 8 10 12

Weeks

# D


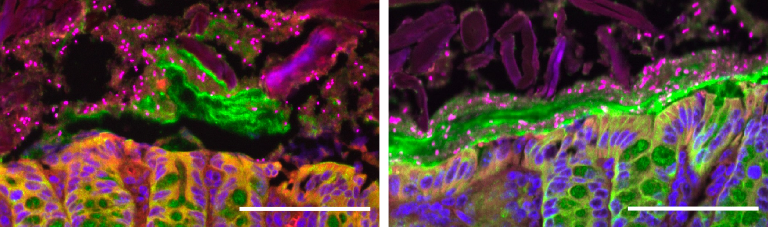


Water

CMC


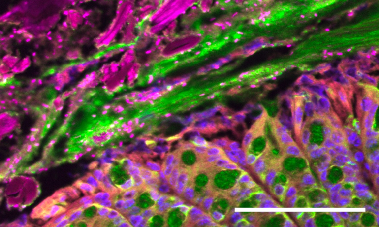


P80

**Figure S3:** Germ-free mice transplanted with mucus-associated microbiota exhibit alterations in their intestinal microbiota composition and localization.
